# Supplementary material for: SARS-CoV-2 infection activates inflammatory macrophages in vascular immune organoids
Source: Sci Rep. 2024 Apr 16;14:8781. doi: 10.1038/s41598-024-59405-9 (PMC11021416; doi:10.1038/s41598-024-59405-9)
Supplement: Supplementary file 1 — Supplementary Figures. [file 41598_2024_59405_MOESM1_ESM.pptx]

## Slide 1
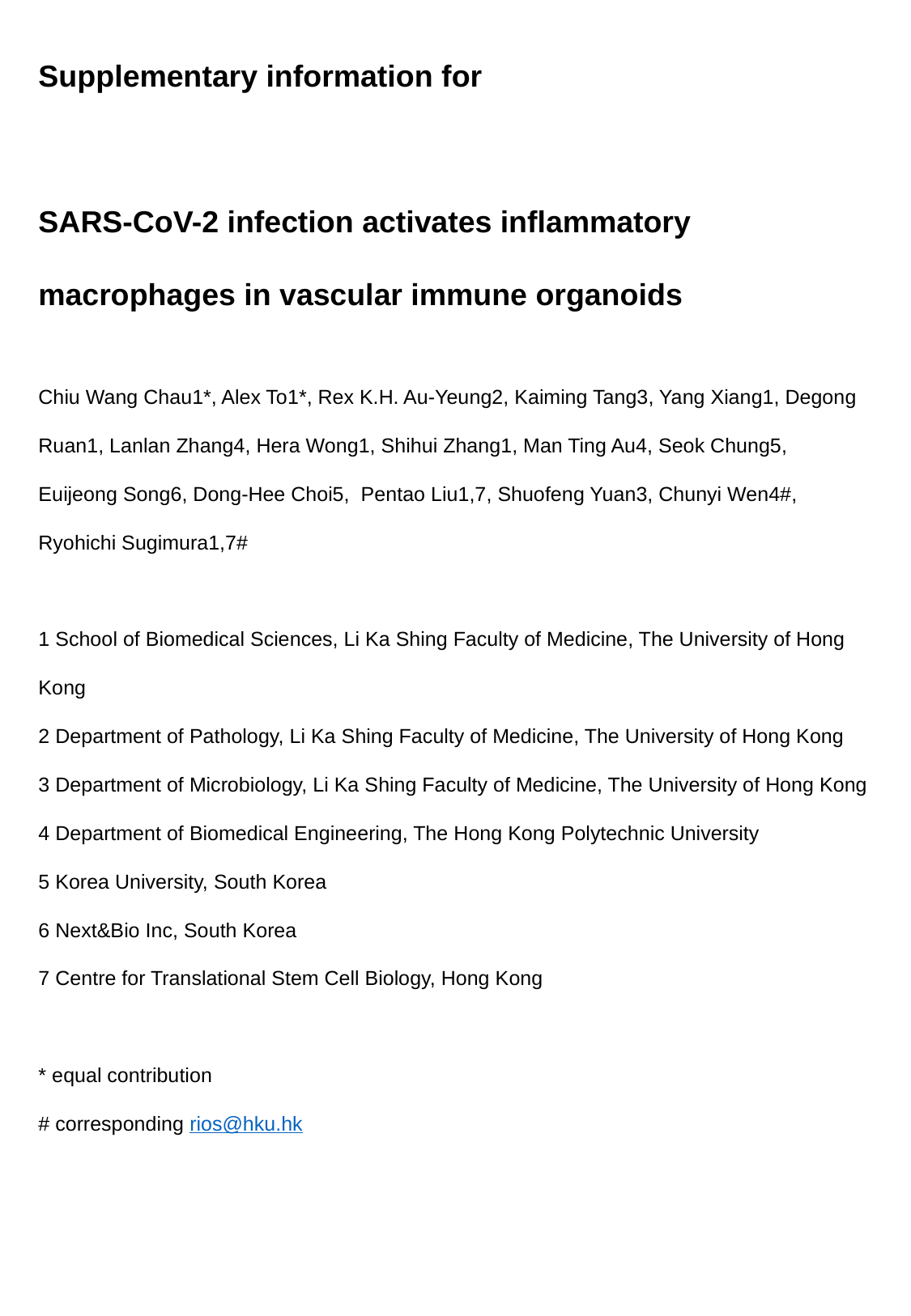

Supplementary information for
SARS-CoV-2 infection activates inflammatory macrophages in vascular immune organoids
Chiu Wang Chau1*, Alex To1*, Rex K.H. Au-Yeung2, Kaiming Tang3, Yang Xiang1, Degong Ruan1, Lanlan Zhang4, Hera Wong1, Shihui Zhang1, Man Ting Au4, Seok Chung5, Euijeong Song6, Dong-Hee Choi5, Pentao Liu1,7, Shuofeng Yuan3, Chunyi Wen4#, Ryohichi Sugimura1,7#
1 School of Biomedical Sciences, Li Ka Shing Faculty of Medicine, The University of Hong Kong
2 Department of Pathology, Li Ka Shing Faculty of Medicine, The University of Hong Kong
3 Department of Microbiology, Li Ka Shing Faculty of Medicine, The University of Hong Kong
4 Department of Biomedical Engineering, The Hong Kong Polytechnic University
5 Korea University, South Korea
6 Next&Bio Inc, South Korea
7 Centre for Translational Stem Cell Biology, Hong Kong
* equal contribution
# corresponding rios@hku.hk

## Slide 2
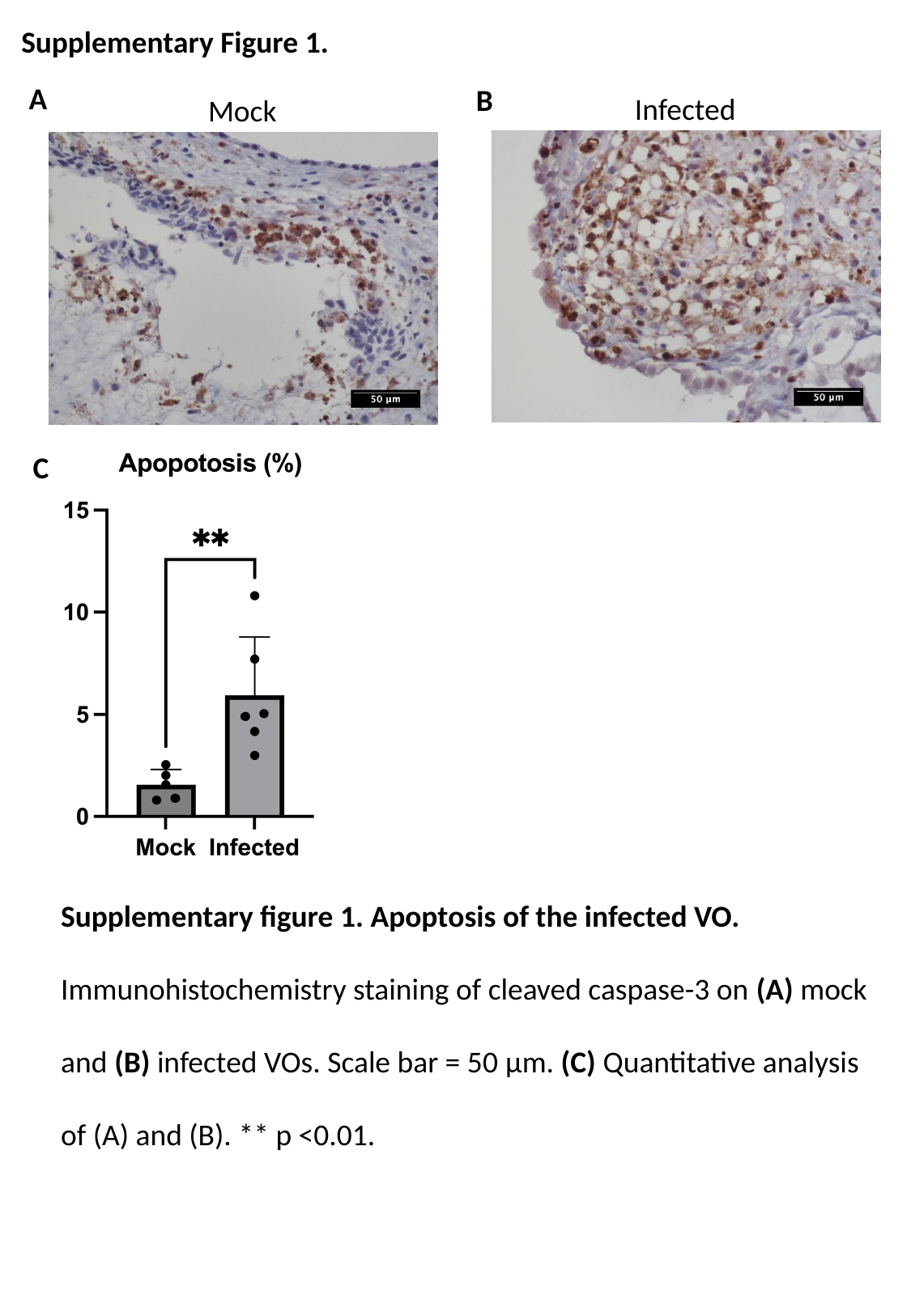

Supplementary Figure 1.
A
B
Infected
Mock
C
Supplementary figure 1. Apoptosis of the infected VO. Immunohistochemistry staining of cleaved caspase-3 on (A) mock and (B) infected VOs. Scale bar = 50 μm. (C) Quantitative analysis of (A) and (B). ** p <0.01.

## Slide 3
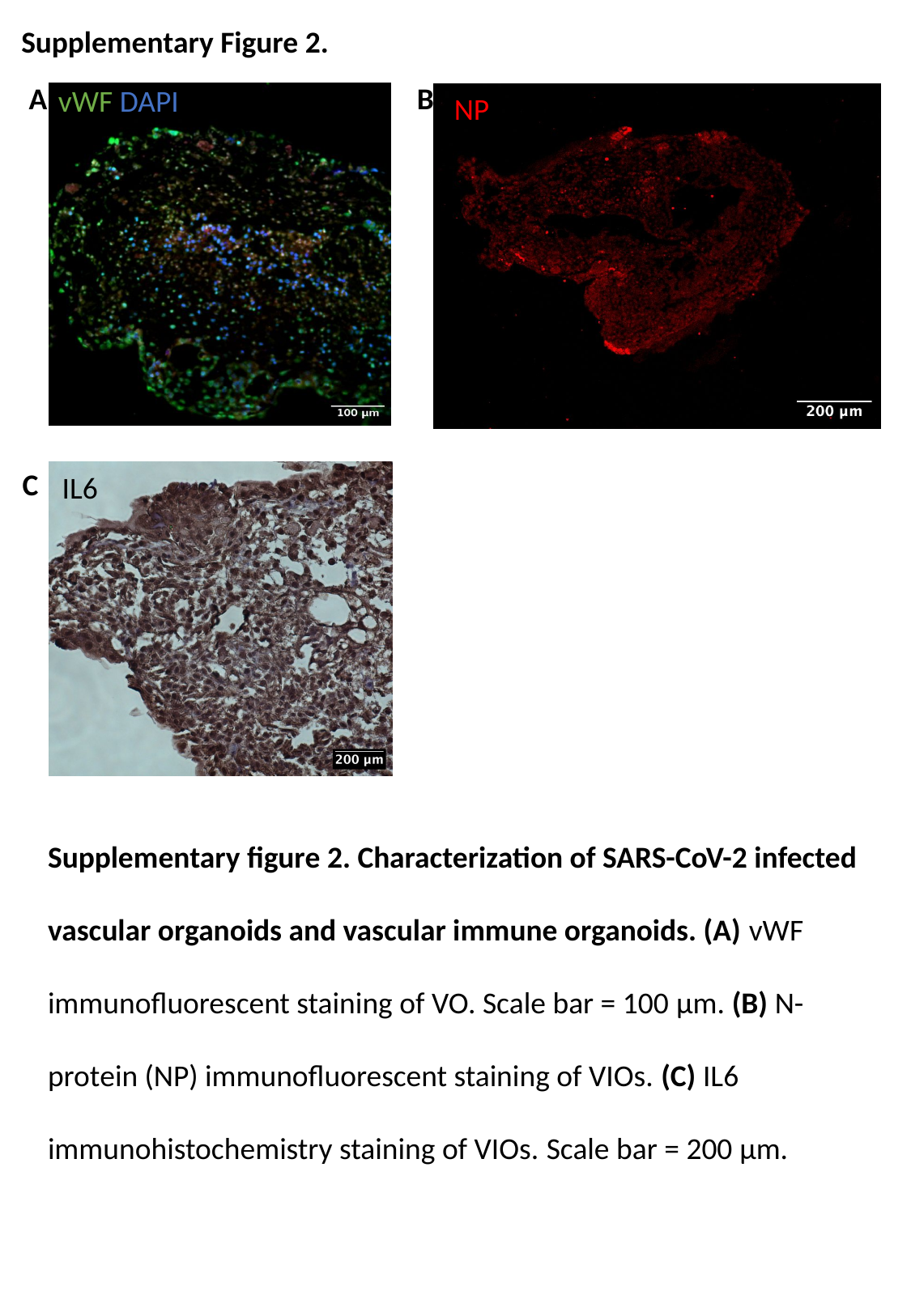

Supplementary Figure 2.
B
A
vWF DAPI
NP
C
IL6
Supplementary figure 2. Characterization of SARS-CoV-2 infected vascular organoids and vascular immune organoids. (A) vWF immunofluorescent staining of VO. Scale bar = 100 μm. (B) N-protein (NP) immunofluorescent staining of VIOs. (C) IL6 immunohistochemistry staining of VIOs. Scale bar = 200 μm.

## Slide 4
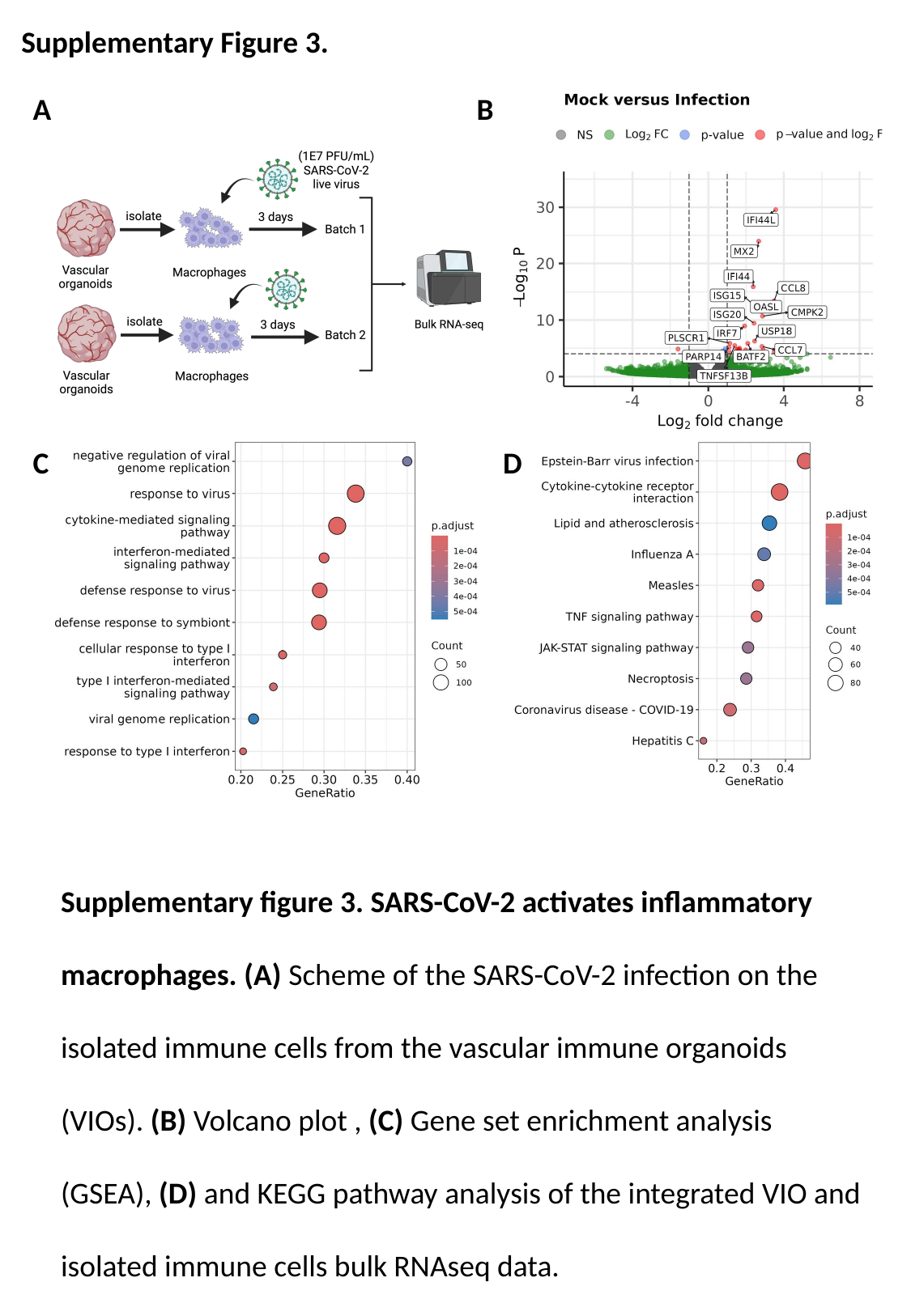

Supplementary Figure 3.
A
B
C
D
Supplementary figure 3. SARS-CoV-2 activates inflammatory macrophages. (A) Scheme of the SARS-CoV-2 infection on the isolated immune cells from the vascular immune organoids (VIOs). (B) Volcano plot , (C) Gene set enrichment analysis (GSEA), (D) and KEGG pathway analysis of the integrated VIO and isolated immune cells bulk RNAseq data.

## Slide 5
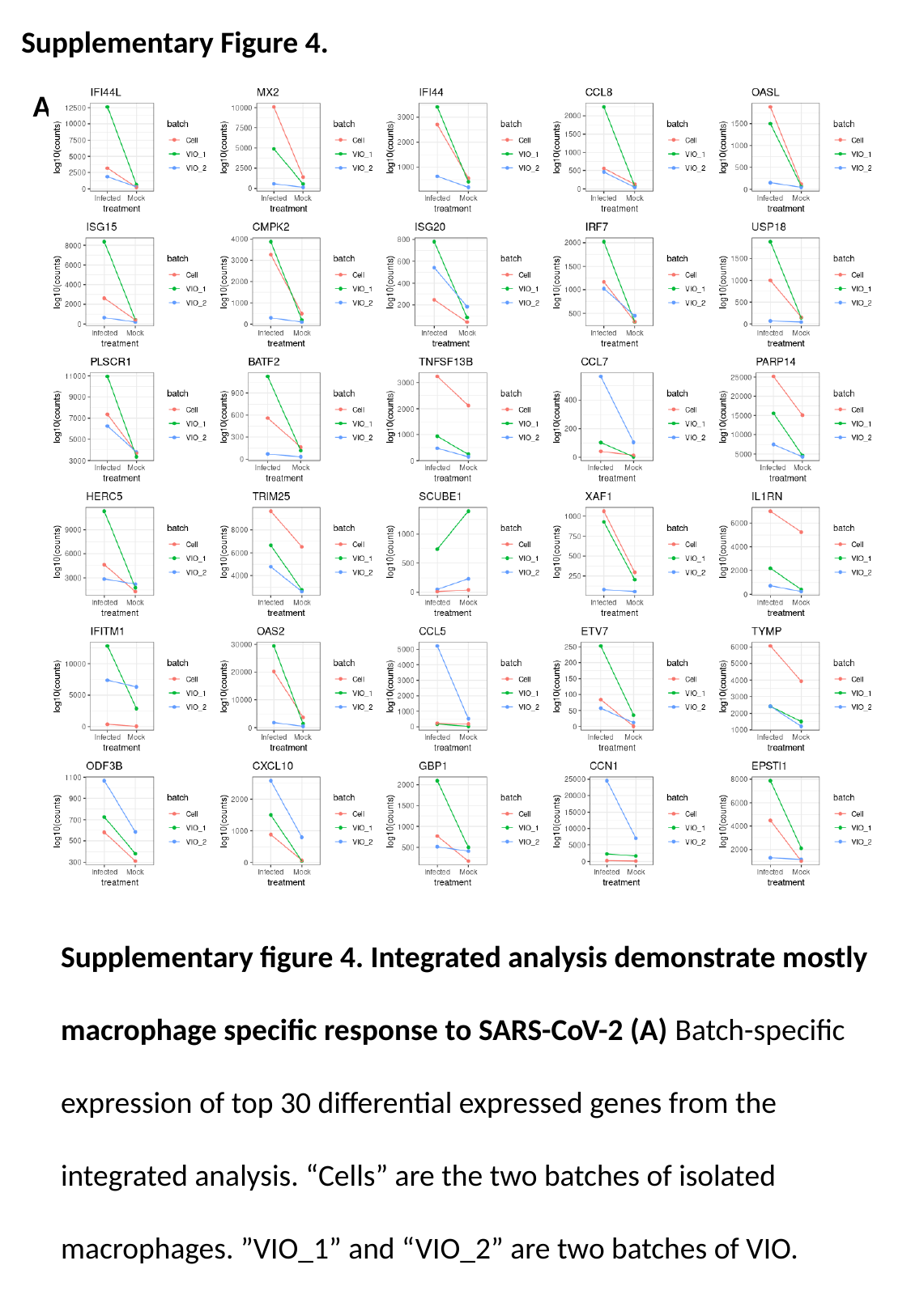

Supplementary Figure 4.
A
Supplementary figure 4. Integrated analysis demonstrate mostly macrophage specific response to SARS-CoV-2 (A) Batch-specific expression of top 30 differential expressed genes from the integrated analysis. “Cells” are the two batches of isolated macrophages. ”VIO_1” and “VIO_2” are two batches of VIO.
